# The prevalence of and factors associated with high-risk alcohol consumption in Korean adults: The 2009–2011 Korea National Health and Nutrition Examination Survey

**DOI:** 10.1371/journal.pone.0175299

**Published:** 2017-04-06

**Authors:** Jae Won Hong, Jung Hyun Noh, Dong-Jun Kim

**Affiliations:** Department of Internal Medicine, Ilsan-Paik Hospital, College of Medicine, Inje University, Koyang, Gyeonggi-do, Republic of Korea; University of Louisville School of Medicine, UNITED STATES

## Abstract

**Background:**

The consequences of alcohol consumption on health outcomes are largely determined by two separate, but related, dimensions of drinking: the total volume of alcohol consumed and the pattern of drinking. Most epidemiological studies focus on the amount of alcohol consumed and do not consider the pattern of drinking.

**Objectives:**

This study evaluated the prevalence of and factors associated with high-risk and heavy alcohol drinking in Korean adults.

**Methods:**

This study analyzed 15,215 of the 28,009 participants in the 2009–2011 Korea National Health and Nutrition Examination Survey (KNHANES). High-risk alcohol drinking was defined as Alcohol Use Disorders Identification Test (AUDIT) scores ≥16, which provides a framework for intervention to identify hazardous and harmful drinking patterns as the cause of alcohol-use disorders, according to World Health Organization guidelines.

**Results:**

The prevalence of high-risk drinking was 15.1%, with the highest prevalence of 17.2% in middle-aged adults (45–64 years). In men, the prevalence of high-risk alcohol drinking was 23.7%, with the highest prevalence found in middle-aged adults. In women, the prevalence of high-risk alcohol drinking was 4.2%, with the highest prevalence found in younger adults. Men had higher weighted mean AUDIT scores than women (10.0 *vs*. 4.0, *P*<0.001), and age was negatively associated with the AUDIT score (*P*<0.001). Elementary school graduates had higher mean AUDIT scores than senior high school (*P* = 0.003) or college (*P*<0.001) graduates. Regarding occupation, clerical support workers (*P* = 0.002) and service and sales workers (*P*<0.001) had higher mean AUDIT scores than managers and professionals. Logistic regression analyses of high-risk alcohol drinking using sex, age, education level, number of family members, household income, and occupation as covariates was performed. Women had a lower risk of high-risk alcohol drinking (odds ratio (OR) 0.14, 95% CI: 0.13–0.16, *P*<0.001) than men. Regarding age, compared to control subjects aged 19–29 years, adults aged 60–69 and older than 70 years had 0.67- (95% CI: 0.51–0.89, *P* = 0.005) and 0.29-fold (95% CI: 0.20–0.70, *P*<0.001) lower risks, respectively, of high-risk alcohol drinking, whereas adults aged 30–59 had an increased risk of high-risk alcohol drinking. Using elementary school graduates as controls, senior high school (OR: 0.70, 95% CI: 0.55–0.87, *P* = 0.002) and college (OR: 0.54, 95% CI: 0.42–0. 70, *P*<0.001) graduates had lower risks of high-risk alcohol drinking. Regarding occupation, compared to managers and professionals as controls, service and sales workers had a greater risk of high-risk alcohol drinking (OR: 1.36, 95% CI: 1.07–1.73, *P* = 0.011). The number of family members and household income did not influence high-risk alcohol drinking.

**Conclusions:**

In a representative sample of Korean adults, the prevalence of high-risk alcohol drinking was 15.1%, with the highest prevalence of 28.3% found in middle-aged men (45–64 years). This study suggests that younger age, male sex, low education level, and service and sales workers are at risk for a high-risk drinking pattern.

## Introduction

Alcohol consumption is major risk factor for burden of disease, particularly bouts of heavy drinking. Diseases in which alcohol has a detrimental effect include unintentional or intentional injuries, cancer, liver cirrhosis, cardiovascular diseases, diabetes mellitus, and neuropsychiatric disorders, with an estimated 3.8% of all global deaths and 4.6% of global disability-adjusted life-years attributable to alcohol [1].

Based on the World Health Organization (WHO) Global Status Report on Alcohol and Health 2014, an average of 6.13 L pure alcohol (defined as 100% ethanol) was consumed worldwide each year in individuals aged 15 years or older [2]. The countries with the highest overall consumption were in Eastern Europe (≥12 L pure alcohol per individual). Korea was the region with the next highest overall consumption (8.5–11.9 L pure alcohol per individual) in 2015 [3].

However, there is discordance between the amount of alcohol consumed and the prevalence of alcohol-use disorders. Unlike the average volume of alcohol consumed per adult, the highest prevalence of alcohol-use disorders was in Southeast Asia, America, and the western Pacific region [1]. This finding can be partly explained by hazardous drinking patterns that lead to harmful consequences. The countries with the highest prevalence of heavy episodic drinking (at least 60 g pure alcohol on at least one occasion in the past 7 days) among current drinkers were in Southeast Asia and Mongolia (≥30%) [4].

Most epidemiology studies focus on the amount of alcohol consumed, and few considering the pattern of drinking. Therefore, we evaluated high-risk alcohol consumption using the Alcohol Use Disorders Identification Test (AUDIT), which provides a framework for intervention to identify hazardous and harmful drinking patterns as the cause of alcohol-use disorders, as well as heavy alcohol drinking [5].

In the current study, we performed a cross-sectional analysis to investigate the prevalence of and factors associated with high-risk alcohol drinking, using AUDIT, in Korean adults based on data from the 2009–2011 Korean National Health and Nutrition Examination Survey (KNHANES).

## Methods

### Study population and data collection

This study used data from the 2009–2011 KNHANES, a cross-sectional, nationally representative survey conducted by the Korean Center for Disease Control for Health Statistics. The following information is reproduced from our previous works [6–8]. The KNHANES has been conducted periodically since 1998 to assess the health and nutritional status of the civilian, non-institutionalized population of Korea. Participants were selected using proportional allocation-systemic sampling with multistage stratification. A standardized interview was conducted in the homes of the participants to collect information on demographic variables, family and medical history, medications used, and a variety of other health-related variables. The health interview used an established questionnaire to determine the demographic and socioeconomic characteristics of the subjects including age, education level, occupation, household income, marital status, smoking habit, alcohol consumption, exercise, previous and current diseases, and family disease history.

### The assessment of alcohol consumption

Alcohol consumption was assessed by questioning the subjects about their drinking behavior, including the average amount consumed and drinking frequency, in the month before the interview. As described in detail previously [9,10], a standard drink was defined as a single glass of liquor, wine, or the Korean traditional distilled liquor So-ju. One bottle of beer (355 mL) was counted as 1.6 standard drinks. We calculated the amount of alcohol consumed per standard drink to be 10 g, and the average daily alcohol intake was assessed [9,10]. An average consumption of 30 g per day or more, a level of exposure associated with health risks, was considered heavy alcohol drinking [9,11–14].

### AUDIT

To assess high-risk alcohol drinking in this study, we used the AUDIT, which was developed by the WHO as a simple method of screening for excessive drinking [5]. The AUDIT comprises three domains: hazardous alcohol use (frequency of drinking, typical quantity, and frequency of heavy drinking), dependence symptoms (impaired control over drinking, increased salience of drinking, and morning drinking), and harmful alcohol use (guilt after drinking, blackouts, alcohol-related injuries, and other concerns about drinking). The AUDIT scores were categorized into three groups according to the WHO guidelines: low-risk, 0 to 7 points; intermediate-risk, 8 to 15 points; and high-risk, ≥16 points. We found that with AUDIT scores of 8 to 15 it was most appropriate to provide simple advice focused on a reduction in hazardous drinking or medium-level alcohol problems, whereas AUDIT scores ≥16 represented high-risk alcohol drinking, suggesting the need for counseling and continued monitoring or further diagnostic evaluation for alcohol dependence [5].

### Ethics statement

This study was approved by the Institutional Review Board of Ilsan Paik Hospital, Republic of Korea (IRB 2016-12-022). After approving the study proposal, the KNHANES dataset was made available at the request of the investigator. Our study was exempt from participant consent because the dataset did not include any personal information and the participants’ consent had already been given for the KNHANES.

### Statistical analyses

The KNHANES participants were not sampled randomly. The survey was designed using a complex, stratified, multistage probability-sampling model; consequently, individual participants were not equally representative of the Korean population. To obtain representative prevalence rates from the dataset, it is necessary to consider the power of each participant (sample weight) as representative of the Korean population. Following approval from the Korea Centers for Disease Control and Prevention, we received a survey dataset that included information regarding the survey location, strata by age, sex, and various other factors, and the sample weight for each participant. The survey sample weights, which were calculated using the sampling and response rates and age/sex proportions of the reference population (2005 Korean National Census Registry), were used in all of the analyses to provide representative estimates of the non-institutionalized Korean civilian population. The statistical analyses were performed using SPSS ver. 21.0 for Windows (SPSS, Chicago, IL, USA). To compare the weighted mean AUDIT score according to socio-demographic factors, the chi-square test and analysis of covariance (ANCOVA) were performed. A logistic regression analysis was used to evaluate the odds ratio (OR) for heavy alcohol drinking and high-risk drinking with age, sex, education level, number of family members, household income, and occupation as covariates. All of the tests were two-sided, and *P* values < 0.05 were considered statistically significant.

## Results

### Demographics and clinical characteristics of the study population

Among the 28,009 participants in the 2009–2011 KNHANES, 6,810 individuals younger than 19 years of age were excluded. The 3,199 adults who did not undergo blood collection were excluded, as were the 2,785 subjects who lacked AUDIT scores. Ultimately, this study analyzed 15,215 participants. Table 1 shows the weighted demographic and clinical characteristics of the study population. The mean AUDIT score was 2 points. The percentages of subjects with low-, intermediate-, and high-risk AUDIT scores were 60.5%, 24.4%, and 15.1%, respectively. The weighted average alcohol intake was 20.1 (95% CI: 19.4–20.9) g/day. The overall weighted prevalence of heavy alcohol consumption (alcohol ≥ 30 g/day) was 21.7% (95% CI: 20.8–22.6%). The prevalence of heavy alcohol drinkers was much higher in men than in women (33.6% *vs*. 6.6%). The overall weighted prevalence of abstainers was 13.4%, with a higher prevalence in women than in men (19.3% *vs*. 8.7%).

**Table 1:** Demographic and clinical characteristics of the study population.

		Unweighted Number (%)	Weighted Number (%)
Total		15,215	29,850,472
Sex	Men	7,615 (50.0)	16,745,929 (56.1)
	Women	7,600 (50.0)	13,104,543 (43.9)
Age (years)	19–29	2,033 (13.4)	6,116,551 (20.5)
	30–39	3,136 (20.6)	6,696,234 (22.4)
	40–49	3,069 (20.2)	6,831,926 (22.9)
	50–59	2,894 (19.0)	5,399,647 (18.1)
	60–69	2,383 (15.7)	2,902,827 (9.7)
	≥ 70	1,700 (11.2)	1,903,239 (6.4)
Education	Elementary school graduated	3,371 (22.2)	4,816,540 (16.1)
	Junior high school graduated	1,713 (11.3)	3,090,942 (10.4)
	Senior high school graduated	5,426 (35.7)	11,975,403 (40.1)
	College graduated	4,705 (30.9)	9,967,587 (33.4)
Family member (n)	1	917 (6.0)	1,584,546 (5.3)
	2	3,637 (23.9)	5,649,241 (18.9)
	3	3,763 (24.7)	7,875,577 (26.4)
	≥ 4	6,898 (45.3)	14,741,108 (49.4)
Household income	≤ 24^th^ percentile	2,717 (18.1)	4,417,455 (14.8)
	25-49^th^ percentile	3,742 (24.6)	7,674,246 (25.7)
	50-74^th^ percentile	4,356 (28.6)	8,939,547 (29.9)
	≥ 75^th^ percentile	4,370 (28.7)	8,819,224 (29.5)
Occupation	Managers and professionals	1,978 (13.0)	4,393,642 (14.7)
	Clerical support workers	1,346 (8.8)	2,992,076 (10.0)
	Service and sales workers	2,012 (13.2)	4,365,819 (14.6)
	Skilled agricultural, forestry and fishery workers	1,274 (8.4)	1,867,003 (6.3)
	Craft, plant, or machine operators and assemblers	1,664 (10.9)	3,907,122 (13.1)
	Laborers	1,295 (8.5)	2,406,266 (8.1)
	Unemployed (including students and housewives)	5,646 (37.1)	9,918,544 (33.2)

### Weighted prevalence of high-risk and intermediate- or high-risk alcohol drinking according to age group

Table 2 shows the weighted prevalence of high-risk (AUDIT score ≥ 16) and intermediate- or high-risk alcohol drinking (AUDIT score ≥ 8) in Korean adults. Overall, the prevalence of high-risk drinking was 15.1%, with the highest prevalence of 17.2% found in middle-aged adults (45–64 years). The prevalence of intermediate- or high-risk alcohol drinking was 39.5%, with the highest prevalence of 43.9% found in younger adults (19–44 years). In men, the prevalence of high-risk and intermediate- or high-risk was 23.7 and 57.5%, respectively, with the highest prevalence found in middle-aged adults. In women, the prevalence of high-risk and intermediate- or high-risk was 4.2 and 16.6%, with the highest prevalence found in younger adults.

**Table 2:** Weighted prevalence of high-risk and intermediate- or high-risk alcohol drinking according to age group.

	Number (unweighted/weighted)	Prevalence of high risk drinking (AUDIT score ≥ 16)	Prevalence of intermediate or high risk drinking (AUDIT score ≥ 8)
Both men and women			
Total	15,215/29,850,472	15.1 (14.4–15.9)	39.5 (38.5–40.6)
Younger adults	5,169/12,812,835	14.8 (13.5–16.0)	43.9 (42.2–45.5)
Middle-aged adults	7,258/13,896,354	17.2 (16.1–18.3)	39.5 (38.2–40.9)
Older adults	2,788/3,141,283	7.4 (6.1–8.8)	22.0 (20.1–24.0)
Men			
Total	7,615/16,745,929	23.7 (22.4–25.0)	57.5 (56.1–58.9)
Younger adults	2,447/7,261,239	21.3 (19.4–23.2)	59.7 (57.4–62.0)
Middle-aged adults	3,598/7,759,158	28.3 (26.4–30.1)	60.1 (58.2–62.0)
Older adults	1,570/1,725,532	13.1 (10.8–15.4)	36.7 (33.6–39.7)
Women			
Total	7,600/13,104,543	4.2 (3.6–4.9)	16.6 (15.5–17.7)
Younger adults	2,722/5,551,596	6.3 (5.0–7.5)	23.1 (21.0–25.3)
Middle-aged adults	3,660/6,137,196	3.2 (2.5–3.9)	13.5 (12.1–14.9)
Older adults	1,218/1,415,751	0.5 (0.2–0.9)	4.2 (2.9–5.5)

Data are expressed as mean (95% CI). AUDIT, Alcohol Use Disorders Identification Test. Younger adults (age, 19–44 years), Middle-aged adults (age, 45–64 years), Older adults (age ≥ 65 years).

### Weighted mean AUDIT scores according to socio-demographic factors

**Fig 1:**
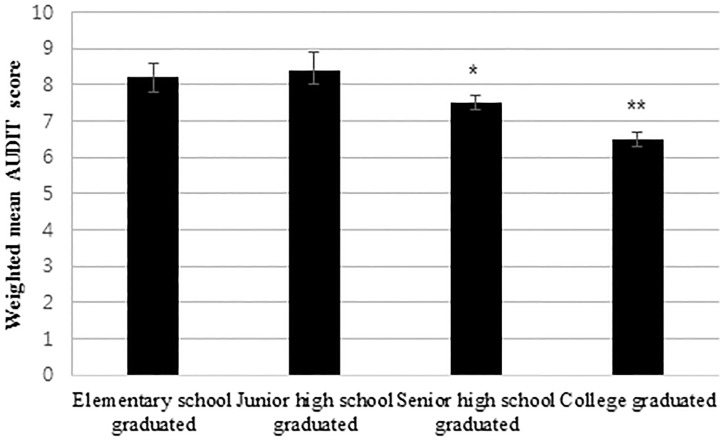
Weighted mean AUDIT (Alcohol Use Disorders Identification Test) scores according to education level. Elementary school graduates had higher mean AUDIT scores than senior high school (*P <0.05) or college graduates (**P<0.001) after adjusting for age, sex, number of family members, household income, and occupation.

**Fig 2:**
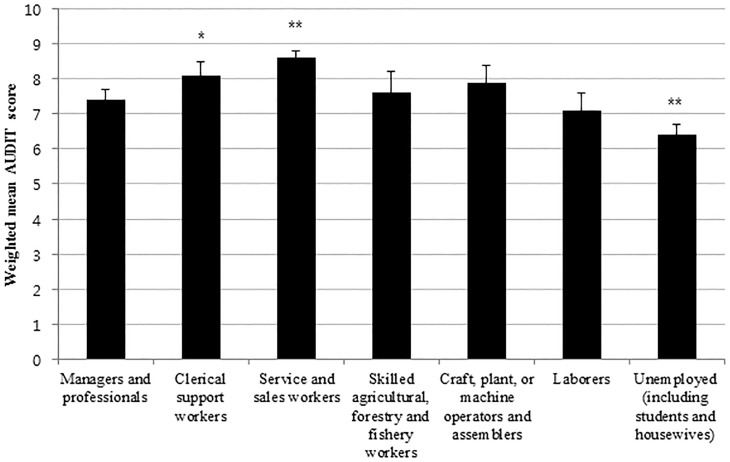
Weighted mean AUDIT (Alcohol Use Disorders Identification Test) scores according to occupation. Clerical support workers (*P<0.005) and service and sales workers (**P<0.001) had higher mean AUDIT scores than managers and professionals, after adjusting for age, sex, number of family members, education level, household income.

Table 3 shows the unadjusted and adjusted-weighted mean AUDIT scores according to socio-demographic factors. Men had a higher weighted mean AUDIT score than women (10.0 *vs*. 4.0, *P*<0.001). According to age, the mean AUDIT score was highest in individuals aged 19–29 years and lowest in those aged ≥ 70 years. Age was negatively associated with the AUDIT score (*P*<0.001). Elementary school graduates had higher mean AUDIT scores than senior high school (*P* = 0.003) or college (*P*<0.001) graduates (Fig 1). Concerning occupation, clerical support workers (*P* = 0.002) and service and sales workers (*P*<0.001) had higher mean AUDIT scores than managers and professionals (Fig 2). The number of family members or household income did not influence the mean AUDIT scores after adjusting for all of the variables.

**Table 3:** Weighted mean AUDIT scores according to socio-demographic factors.

		Unadjusted		Adjusted for all variables	
		AUDIT score, mean (95%CI)	*P*	AUDIT score, mean (95%CI)	*P*
Total		2 (0–36)			
Sex	Men	10.1 (9.8–10.3)	reference	10.0(9.8–10.2)	reference
	Women	3.9 (3.8–4.1)	<0.001	4.0 (3.8–4.1)	<0.001
Age (years)			<0.001		<0.001
	19–29	8.1 (7.7–8.4)	reference	8.3 (8.0–8.7)	reference
	30–39	7.8 (7.5–8.1)	<0.001	8.2 (7.9–8.5)	<0.001
	40–49	7.8 (7.5–8.1)	<0.001	7.9 (7.6–8.2)	<0.001
	50–59	7.5 (7.2–7.9)	<0.001	7.2 (6.9–7.5)	<0.001
	60–69	5.9 (5.5–6.2)	<0.001	5.3 (4.9–5.6)	<0.001
	≥ 70	3.9 (3.5–4.2)	<0.001	3.3 (2.9–3.7)	<0.001
Education			<0.001		<0.001
	Elementary school graduated	5.7 (5.3–6.0)	reference	8.2 (7.8–8.6)	reference
	Junior high school graduated	7.5 (7.0–8.0)	<0.001	8.4 (8.0–8.9)	0.427
	Senior high school graduated	7.9 (7.7–8.1)	<0.001	7.5 (7.3–7.7)	0.003
	College graduated	7.5 (7.3–7.7)	<0.001	6.5 (6.3–6.7)	<0.001
Family member (n)			0.004		0.043
	1	7.2 (6.5–7.8)	reference	7.4 (7.0–7.9)	reference
	2	7.0 (6.7–7.4)	0.752	7.5 (7.1–7.8)	0.931
	3	7.1 (6.9–7.4)	0.990	7.1 (6.8–7.3)	0.157
	≥ 4	7.7 (7.4–7.9)	0.137	7.5 (7.3–7.7)	0.858
Household income			<0.001		0.907
	≤ 24^th^ percentile	6.5 (6.1–6.9)	reference	7.3 (6.9–7.6)	reference
	25-49^th^ percentile	7.3 (7.0–7.6)	<0.001	7.3 (7.0–7.6)	0.798
	50-74^th^ percentile	7.7 (7.4–7.9)	<0.001	7.4 (7.2–7.7)	0.512
	≥ 75^th^ percentile	7.6 (7.3–7.8)	<0.001	7.4 (7.2–7.7)	0.552
Occupation			<0.001		<0.001
	Managers and professionals	7.9 (7.5–8.3)	reference	7.4 (7.0–7.7)	reference
	Clerical support workers	8.8 (8.4–9.2)	<0.001	8.1 (7.7–8.5)	0.002
	Service and sales workers	8.5 (8.1–8.9)	0.028	8.6 (8.2–8.9)	<0.001
	Skilled agricultural, forestry and fishery workers	7.7 (7.0–8.4)	0.689	7.6 (6.9–8.2)	0.584
	Craft, plant, or machine operators and assemblers	10.0 (9.6–10.5)	<0.001	7.9 (7.5–8.4)	0.059
	Laborers	6.6 (6.1–7.1)	<0.001	7.1 (6.7–7.6)	0.480
	Unemployed (including students and housewives)	5.3 (5.1–5.5)	<0.001	6.4 (6.2–6.7)	<0.001

AUDIT, Alcohol Use Disorders Identification Test.

### Factors associated with high-risk, intermediate- or high-risk alcohol drinking, and heavy alcohol drinking

A logistic regression analysis was performed for high-risk and intermediate- or high-risk alcohol drinking/heavy alcohol drinking using sex, age, education level, number of family members, household income, and occupation as covariates (Table 4). Women had a lower risk of high-risk alcohol drinking (OR: 0.14, 95% CI: 0.13–0.16, *P*<0.001) than men. Regarding age, using subjects aged 19–29 years as controls, adults aged 60–69 and older than 70 years had 0.67- (95% CI: 0.51–0.89, *P* = 0.005) and 0.29-fold (95% CI: 0.20–0.70, *P*<0.001) lower risks of high-risk alcohol drinking, respectively. However, adults aged 30–59 had increased risk of high-risk alcohol drinking compared to those aged 19–29 years. Unlike high-risk alcohol drinking, intermediate- or high-risk alcohol drinking significantly decreased with age (*P*<0.001). Using elementary school graduates as a control, senior high school (OR: 0.70, 95% CI: 0.55–0.87, *P* = 0.002) and college (OR: 0.54, 95% CI: 0.42–0.70, *P*<0.001) graduates had a decreased risk of high-risk alcohol drinking. The ORs of intermediate- or high-risk alcohol drinking were similar to those of high-risk alcohol drinking, according to education level. Regarding occupation, using managers and professionals as a control, service and sales workers had increased risks of high-risk (OR: 1.36, 95% CI: 1.07–1.73, *P* = 0.011) and intermediate or high-risk (OR: 1.45, 95% CI: 1.23–1.71, *P*<0.001) alcohol drinking. Conversely, unemployed status, including students and housewives, was associated with decreased risks of high-risk (OR: 0.77, 95% CI: 0.60–0.98, *P* = 0.032) and intermediate- or high-risk (OR: 0.76, 95% CI: 0.64–0.90, *P* = 0.001) alcohol drinking. The number of family members and household income did not influence high-risk alcohol drinking. The factors associated with heavy alcohol drinking, based on an average of 30 g alcohol per day or more, and high-risk alcohol drinking, using the AUDIT score, were similar, with the exception of the number of family members. Using living alone as a control, three family members were associated with a decreased risk of heavy alcohol drinking (OR: 0.70, 95% CI: 0.53–0.93, *P* = 0.015).

**Table 4:** Odds ratios (ORs) for heavy alcohol drinking (≥ 30 g/day), high-risk drinking (AUDIT score ≥ 16), and intermediate- or high-risk drinking (AUDIT score ≥ 8).

		Heavy alcohol drinking		High-risk drinking		Intermediate- or high-risk drinking	
Variables		OR (95% CI)	*P*	OR (95% CI)	*P*	OR (95% CI)	*P*
Sex	Men	Reference		Reference		Reference	
	Women	0.14 (0.12–0.16)	<0.001	0.14 (0.12–0.17)	<0.001	0.15 (0.13–0.16)	<0.001
Age (years)			<0.001		<0.001		<0.001
	19–29	Reference		Reference		Reference	0.298
	30–39	1.54 (1.27–1.88)	<0.001	1.39 (1.12–1.71)	0.002	0.92 (0.78–1.08)	0.003
	40–49	1.62 (1.34–1.96)	<0.001	1.39 (1.13–1.71)	0.002	0.79 (0.67–0.92)	<0.001
	50–59	1.54 (1.24–1.91)	<0.001	1.30 (1.02–1.67)	0.037	0.66 (0.56–0.78)	<0.001
	60–69	0.87 (0.69–1.11)	0.267	0.67 (0.51–0.89)	0.005	0.39 (0.32–0.48)	<0.001
	≥ 70	0.53 (0.40–0.72)	<0.001	0.29 (0.20–0.42)	<0.001	0.19 (0.15–0.25)	0.298
Education			<0.001		<0.001		<0.001
	Elementary school graduated	Reference		Reference		Reference	
	Junior high school graduated	0.92 (0.75–1.12)	0.393	0.88 (0.69–1.11)	0.279	0.96 (0.79–1.16)	0.655
	Senior high school graduated	0.73 (0.61–0.87)	<0.001	0.70 (0.55–0.87)	0.002	0.83 (0.71–0.88)	0.025
	College graduated	0.54 (0.44–0.67)	<0.001	0.54 (0.42–0.70)	<0.001	0.68 (0.57–0.81)	<0.001
Family member (n)			0.015		0.061		0.032
	1	Reference		Reference		Reference	
	2	0.91 (0.69–1.20)	0.205	0.93 (0.69–1.26)	0.565	1.01 (0.79–1.28)	0.961
	3	0.70 (0.53–0.93)	0.015	0.77 (0.57–1.05)	0.100	0.82 (0.65–1.02)	0.079
	≥ 4	0.84 (0.65–1.10)	0.489	0.94 (0.70–1.27)	0.697	0.88 (0.71–1.09)	0.235
Household income			0.677		0.488		0.488
	≤ 24^th^ percentile	Reference		Reference		Reference	
	25-49^th^ percentile	1.13 (0.93–1.37)	0.222	0.95 (0.77–1.18)	0.652	0.91 (0.77–1.08)	0.274
	50-74^th^ percentile	1.09 (0.90–1.31)	0.373	0.91 (0.74–1.11)	0.348	0.92 (0.77–1.08)	0.303
	≥ 75^th^ percentile	1.07 (0.88–1.30)	0.494	0.86 (0.69–1.06)	0.163	0.98 (0.82–1.17)	0.832
Occupation			<0.001		<0.001		<0.001
	Managers and professionals	Reference		Reference		Reference	
	Clerical support workers	1.17 (0.96–1.42)	0.130	1.19 (0.97–1.47)	0.097	1.33 (1.13–1.58)	0.001
	Service and sales workers	1.51 (1.25–1.82)	<0.001	1.36 (1.07–1.73)	0.011	1.45 (1.23–1.71)	<0.001
	Skilled agricultural, forestry and fishery workers	1.19 (0.91–1.55)	0.212	1.11 (0.80–1.53)	0.534	1.07 (0.84–1.36)	0.567
	Craft, plant, or machine operators and assemblers	1.15 (0.95–1.39)	0.159	0.98 (0.77–1.23)	0.840	1.05 (0.88–1.26)	0.567
	Laborers	0.97 (0.74–1.26)	0.790	0.83 (0.62–1.12)	0.221	0.94 (0.75–1.17)	0.562
	Unemployed (including students and housewives)	0.81 (0.67–0.98)	0.031	0.77 (0.60–0.98)	0.032	0.76 (0.64–0.90)	0.001

AUDIT, Alcohol Use Disorders Identification Test.

## Discussion

Alcohol drinking is one of the most common social behaviors in Korean adults. The mean amount of alcohol consumed per person is 30.1 g/day for men and 6.6 g/day for women, according to Korean statistical data [15]. For selected high-income countries, a previous report showed that all of the countries spent more than 1% of their gross domestic product (GDP) purchasing power parity (PPP) on alcohol-attributable costs, with the highest GDP PPP spent in the United States (2.7%). Furthermore, for the selected middle-income countries, South Korea spent 3.3% of GDP PPP, more than the United States, with $524 alcohol-attributable costs per head [1,16].

Apart from the volume of alcohol consumed, the pattern of drinking also gives rise to very different health outcomes in population groups with the same level of alcohol consumption, particularly injuries and cardiovascular disease [2]. The higher the risky pattern of drinking, the greater the alcohol-attributable burden of disease [2].

Based on the data from the KNHANES 2009–2011, we used the AUDIT score to calculate that the prevalence of high-risk alcohol drinking in the Korean population was 15.1%, with the highest prevalence of 28.3% found in middle-aged men (45–64 years). This result is similar to the data released by the WHO, which noted a prevalence of heavy episodic drinking of 10.0–19.9% in South Korea [4]. Although we could not directly compare the prevalence of high-risk alcohol drinking in the general population among countries, due to different data collection and analysis methodologies, the prevalence of binge drinking in adults, which is considered a high-risk drinking pattern, was 9% in Hong Kong and 17.1% in the United States [17,18].

Various factors that affect the high-risk patterns of alcohol consumption and the magnitude of alcohol-related problems have been identified at the individual and societal level [19–24]. Our study also suggests that socio-demographic factors, including sex, age, education level, and occupation, are associated with high-risk alcohol drinking. However, the number of family members and household income did not influence high-risk alcohol drinking in this study. When we analyzed the factors associated with heavy alcohol drinking (≥ 30 g/day), the number of family members was also associated with heavy alcohol drinking, along with sex, age, education level, and occupation.

Traditionally, men and younger people more consistently engage in hazardous drinking than women and older people in all regions [25,26]. These findings are concordant with our study. Men had an approximately 7-fold greater risk of high-risk alcohol drinking than women. Young adults aged 19–29 years had a 3-fold greater risk of high-risk alcohol drinking compared to those aged ≥ 70 years.

High-risk alcohol drinking, such as binge drinking, particularly in adolescence, is prevalent and has been recognized as a widespread problem behavior for more than a generation [27]. The European School Survey Project in Alcohol and Other Drugs reported that on average, 61% of the students in the European region had consumed alcohol in the last 30 days and 43% had participated in binge drinking in the same period [28]. The Arkhangelsk Social and Health Assessment showed that the overall prevalence of binge drinking was about 50%, using data from a representative sample of 6^th^–10^th^ grade students (n = 2892) in the public school system in Russia [19]. Tavolacci *et al*. [29] reported that the respective prevalence of binge drinking in the never, occasional, and frequent categories was 34.9%, 51.3%, and 13.8%, respectively, among 3286 college students in France. In our study, the prevalence of high-risk drinking in young adults was 14.8% (95% CI: 13.5–16.0%), which was similar to the prevalence of frequent binge drinking among college students in France, as reported by Tavolacci *et al* [29].

Although household income did not affect high-risk alcohol drinking in our study, the level of education and occupation, reflecting socio-economic status (SES), were associated with high-risk alcohol drinking. In this study, concerning occupation, service and sales workers were the most vulnerable to high-risk alcohol drinking. We speculated that social culture, such as entertaining customers with wining and dining, and job stress dealing with clients were possible factors leading to vulnerability of service and sales workers to high-risk alcohol drinking. Similary, Barnes *et al*. reported that those in sales and related occupations were 6.9 percentage points more likely to binge drink than those in professional occupation [30]. Previous studies have also shown that a low education level and unemployment or a low SES are associated higher heavy drinking or drunkenness [21–23,26]. Parikh *et al*. [24] reported that lower annual income and lack of college education are independently associated with higher rates of binge drinking among elderly Americans. Unlike previous findings, this study showed that unemployed status was associated with a decreased risk of high-risk alcohol drinking, most likely because housewives were included as unemployed.

There are several strengths to our study. First, we examined a large, nationally representative sample of adult Koreans. To the best of our knowledge, few other studies have described a national-level assessment of the demographic characteristics and associated risk factors for high-risk alcohol drinking using AUDIT. Nevertheless, our study had some limitations. Although we adjusted for many confounding factors, residual or hidden confounding variables cannot be excluded, similar to other cross-sectional studies. Second, we assessed alcohol consumption based on a self-reported questionnaire. This could lead to misclassification of actual drinking patterns, because participants may underestimate their alcohol consumption by recall error or intentionally.

In conclusion, in a representative sample of Korean adults, the prevalence of high-risk alcohol drinking was 15.1%, with the highest prevalence of 28.3% found in middle-aged men (45–64 years). This study suggests that young male, low education level, and service and sales workers were vulnerable to high-risk drinking pattern. Factors associated with high-risk alcohol drinking should be considered in policy-based interventions to reduce the high-risk pattern of drinking and related alcohol-attributable disease.

## References

[1] Rehm J, Mathers C, Popova S, Thavorncharoensap M, Teerawattananon Y, Patra J. Global burden of disease and injury and economic cost attributable to alcohol use and alcohol-use disorders. The Lancet. 2009;373: 2223–2233. 10.1016/S0140-6736(09)60746-7 19560604

[2] World Health Organization. Global status report on alcohol and health 2014.

[3] World Health Organization. World: Total alcohol per capita consumption, in litres of pure alcohol, projected estimates, 2015. World Health Statistics 2016.

[4] World Health Organization. World: prevalence of heavy episodic drinking among current drinkers (%), 2010. Global Health Observatory Map Gallery. 2014.

[5] Babor TF, Higgins-Biddle JC, Saunders JB, Monteiro MG. The alcohol use disorders identification test: Guidelines for use in primary care (Second edition). World Health Organization 2001.

[6] Han SY, Oh SW, Hong JW, Yi SY, Noh JH, Lee HR, et al Association of Estimated Glomerular Filtration Rate with Hemoglobin Level in Korean Adults: The 2010–2012 Korea National Health and Nutrition Examination Survey. PLoS One. 2016;11: e0150029–e0150029. 10.1371/journal.pone.0150029 27128634 PMC4851309

[7] Hong JW, Ku CR, Noh JH, Ko KS, Rhee BD, Kim D. Association between the presence of iron deficiency anemia and hemoglobin A1c in Korean adults: the 2011–2012 Korea National Health and Nutrition Examination Survey. Medicine. 2015;94: e825–e825. 25997055 10.1097/MD.0000000000000825 PMC4602861

[8] Hong JW, Jeon JH, Ku CR, Noh JH, Yoo HJ, Kim D. The prevalence and factors associated with hearing impairment in the Korean adults: the 2010–2012 Korea National Health and Nutrition Examination Survey (observational study). Medicine. 2015;94: e611–e611. 25761183 10.1097/MD.0000000000000611 PMC4602472

[9] Hyeon JH, Gwak JS, Hong SW, Kwon H, Oh S, Lee CM. Relationship between bone mineral density and alcohol consumption in Korean men: the Fourth Korea National Health and Nutrition Examination Survey (KNHANES), 2008–2009. Asia Pacific Journal of Clinical Nutrition. 2016;25: 308–315. 10.6133/apjcn.2016.25.2.17 27222414

[10] Hong JW, Noh JH, Kim D. Association between Alcohol Intake and Hemoglobin A1c in the Korean Adults: The 2011–2013 Korea National Health and Nutrition Examination Survey. PLoS One. 2016;11: e0167210–e0167210. 10.1371/journal.pone.0167210 27893805 PMC5125693

[11] Song DS, Chang UI, Choi S, Jung YD, Han K, Ko S, et al Heavy Alcohol Consumption with Alcoholic Liver Disease Accelerates Sarcopenia in Elderly Korean Males: The Korean National Health and Nutrition Examination Survey 2008–2010. PLoS One. 2016;11: e0163222–e0163222. 10.1371/journal.pone.0163222 27655344 PMC5031399

[12] Costa JS, Silveira MF, Gazalle FK, Oliveira S, Hallal PC, Menezes AM, et al [Heavy alcohol consumption and associated factors: a population-based study]. Revista de saúde pública. 2004;38: 284–291. 15122386 10.1590/s0034-89102004000200019

[13] Roerecke M, Rehm J. Alcohol consumption, drinking patterns, and ischemic heart disease: a narrative review of meta-analyses and a systematic review and meta-analysis of the impact of heavy drinking occasions on risk for moderate drinkers. BMC Med. 2014;12: 182 10.1186/s12916-014-0182-6 25567363 PMC4203905

[14] Moreira LB, Fuchs FD, Moraes RS, Bredemeier M, Cardozo S, Fuchs SC, et al Alcoholic beverage consumption and associated factors in Porto Alegre, a southern Brazilian city: a population-based survey. Journal of studies on alcohol. 1996;57: 253–259. 8709583 10.15288/jsa.1996.57.253

[15] Hong S, Linton JA, Shim J, Kang H. High-risk drinking is associated with a higher risk of diabetes mellitus in Korean men, based on the 2010–2012 KNHANES. Alcohol. 2015;49: 275–281. 25920001 10.1016/j.alcohol.2015.02.004

[16] Chung W, Chun H, Lee S. [Socioeconomic costs of alcohol drinking in Korea]. Journal of preventive medicine and public health. 2006;39: 21–29. 16613068

[17] Kim JH, Lee S, Chow J, Lau J, Tsang A, Choi J, et al Prevalence and the factors associated with binge drinking, alcohol abuse, and alcohol dependence: a population-based study of Chinese adults in Hong Kong. Alcohol and alcoholism. 2008;43: 360–370. 10.1093/alcalc/agm181 18230698

[18] Centers for Disease Control and Prevention (CDC). Vital signs: binge drinking prevalence, frequency, and intensity among adults—United States, 2010. MMWR Morb Mortal Wkly Rep. 2012;61: 14–19. 22237031

[19] Stickley A, Koyanagi A, Koposov R, McKee M, Roberts B, Murphy A, et al Binge drinking among adolescents in Russia: prevalence, risk and protective factors. Addictive behaviors. 2013;38: 1988–1995. 10.1016/j.addbeh.2012.12.009 23384452

[20] Caetano R, Vaeth PA, Canino G. Prevalence and predictors of drinking, binge drinking, and related health and social problems in Puerto Rico. American journal on addictions. 2016;25: 478–485. 10.1111/ajad.12418 27495377

[21] Kydd RM, Connor J. Inconsistency in reporting abstention and heavy drinking frequency: associations with sex and socioeconomic status, and potential impacts. Alcohol and alcoholism. 2015;50: 333–345. 10.1093/alcalc/agu106 25648932 PMC4481535

[22] Torikka A, Kaltiala-Heino R, Luukkaala T, Rimpela A. Trends in Alcohol Use among Adolescents from 2000 to 2011: The Role of Socioeconomic Status and Depression. Alcohol Alcohol. 2016. 2016/08/11. 10.1093/alcalc/agw048 27507821

[23] Vladimirov D, Niemelä S, Auvinen J, Timonen M, Keinänen Kiukaanniemi S, Ala Mursula L, et al Changes in alcohol use in relation to sociodemographic factors in early midlife. Scandinavian journal of public health. 2016;44: 249–257. 10.1177/1403494815622088 26685194

[24] Parikh RB, Junquera P, Canaan Y, Oms JD. Predictors of binge drinking in elderly Americans. American journal on addictions. 2015;24: 621–627. 10.1111/ajad.12275 26300301

[25] Naimi TS, Brewer RD, Mokdad A, Denny C, Serdula MK, Marks JS. Binge drinking among US adults. JAMA: the Journal of the American Medical Association. 2003;289: 70–75. 12503979 10.1001/jama.289.1.70

[26] Naimi TS, Nelson DE, Brewer RD. The intensity of binge alcohol consumption among U.S. adults. American journal of preventive medicine. 2010;38: 201–207. 10.1016/j.amepre.2009.09.039 20117577

[27] Elisaus P, Williams G, Bourke M, Clough G, Harrison A, Verma A. Factors associated with the prevalence of adolescent binge drinking in the urban areas of Greater Manchester. Eur J Public Health. 2015. 2015/10/03. 10.1093/eurpub/ckv115 26428481

[28] Hibell B, Guttormasson U, Ahlstrom S. Substance use among students in 35 countries ESPAD. The 2007 ESPAD Report. 2009.

[29] Tavolacci MP, Boerg E, Richard L, Meyrignac G, Dechelotte P, Ladner J. Prevalence of binge drinking and associated behaviours among 3286 college students in France. BMC Public Health. 2016;16: 178 10.1186/s12889-016-2863-x 26905284 PMC4765104

[30] Barnes AJ, Brown ER. Occupation as an independent risk factor for binge drinking. The American journal of drug and alcohol abuse. 2013;39: 108–114. 10.3109/00952990.2012.694537 22746372 PMC5618795

